# The largest reported papillary thyroid carcinoma arising in struma ovarii and metastasis to opposite ovary: case report and review of literature

**DOI:** 10.1186/s13044-018-0054-9

**Published:** 2018-07-24

**Authors:** Mohamed S. Al Hassan, Tamer Saafan, Walid El Ansari, Afaf A. Al Ansari, Mahmoud A. Zirie, Hanan Farghaly, Abdelrahman Abdelaal

**Affiliations:** 1 Department of General Surgery, Hamad General Hospital, Doha, Qatar; 2 Department of Surgery, Hamad General Hospital, Doha, Qatar; 3 College of Medicine, Qatar University, Doha, Qatar; 4 Department of Gynecologic Oncology, Hamad General Hospital, Doha, Qatar; 5 Department of Endocrinology, Hamad General Hospital, Doha, Qatar; 6 Department of Pathology, Hamad General Hospital, Doha, Qatar

**Keywords:** Total abdominal hysterectomy, Oopherectomy, Salipingo-oopherectomy, Thyroid cancer, Malignant struma ovarii, Papillary thyroid carcinoma, Follicular thyroid carcinoma

## Abstract

**Background:**

Malignant struma ovarii (MSO) is a very rare, germ cell tumor of the ovary, histologically identical to differentiated thyroid cancers. Struma ovarii (SO) is difficult to diagnose on clinical basis or imaging and is mostly discovered incidentally, with few published cases in the literature.

**Case presentation:**

A 42-year old primiparous woman presented with abdominal pain and midline pelvic palpable firm mass arising from the pelvis. Imaging showed pelvic solid cystic mass. Total abdominal hysterectomy, bilateral salpingo-oopherectomy (TAH BSO) and infracolic omentectomy were performed. Histopathology revealed left ovary papillary thyroid carcinoma (PTC) arising in SO (11 cm) and metastatic papillary thyroid carcinoma in the right ovary. Thyroid functions tests were all normal, ultrasound thyroid showed two complex nodules in the left thyroid lobe. Total thyroidectomy was decided, but the patient refused further surgical management and was lost to follow up as she left the country. We undertook a comprehensive literature search, and MSO and thyroid management data from 23 additional publications were analyzed and tabulated. This PTC MSO is probably the largest reported in the literature.

**Conclusions:**

Among the different surgeries for MSO, TAH + BSO appears to have the best clinical outcome. However, unilateral salpingo-oopherectomy/ unilateral oophorectomy and bilateral salpingo-oopherectomy also seem effective. Ovarian cystectomy alone seems associated with higher recurrence. There remains no consensus on the associations between MSO tumor size and potential extent of metastasis, and about the management of thyroid gland. However, surveillance and thyroid gland work up to detect concurrent thyroid cancer are recommended.

## Background

Struma ovarii (SO) is a specialized or monodermal teratoma predominantly composed of mature thyroid tissue (thyroid tissue must comprise > 50% of overall tissue) [1]. SO accounts for ≈5% of all ovarian teratomas [2–4]. Histologically, SO can be benign or malignant [5], although malignant struma ovarii (MSO) is rare (< 5% of cases), and metastasis is rare (0.3–0.5%) [6, 7]. SO is difficult to diagnose on basis of clinical manifestations or imaging, and most cases are incidental findings in patients aged 40–60 years, with a mean age of diagnosis of 43 years [4, 5, 8].

Common presenting symptoms include abdominal pain (20.6%), palpable lower abdominal mass (23.5%), vaginal bleeding (8.8%), or asymptomatic (41.2%, tumor discovered by routine ultrasound). Tachycardia and ascites are sometimes present (12%, 16% of patients respectively). Clinical and biochemical features of hyperthyroidism are uncommon in women with SO (< 5–8% of cases) [3, 5, 6], and whilst some reports observed no SO patients with overt hyperthyroidism symptoms (hence no thyroid function tests undertaken), others found 5–8% incidence of hyperthyroidism with SO [4, 9, 10]. SO women with hyperthyroidism can also have goiter and/or Grave’s disease, but the incidence is very rare [11, 12]. Seldom, seeding of the peritoneum by a benign tumor can occur (strumosis), which may present with ascites with or without pleural effusion [13, 14].

As for imaging, ultrasound appearance of SO may be as heterogeneous uni/multilocular solid mass or multilocular cystic masses [15–17]. An ultrasound feature of SO is the presence of one or more well circumscribed roundish areas of solid tissue with smooth surface ‘struma pearls’, often vascularized at Doppler examination, but otherwise similar (but not identical) to the ‘white ball’ comprising hair and sebum usually seen at ultrasound of dermoid cysts [17].

In women presenting with a pelvic mass, SO is typically diagnosed postoperatively based upon histologic findings of thyroid follicles in the resected ovary, where the histological pattern may show micro/macrofollicular or oxyphil adenoma, with/without papillary hyperplasia [12, 18]. As in thyroid gland follicular tumors, the thyroid epithelium in the teratoma may be organized in a solid, embryonal or pseudotubular pattern, rather than thyroid follicles [19].

We present an extraordinary case of a primiparous woman with large SO containing papillary thyroid carcinoma, with metastasis to the contralateral ovary. To the best of our knowledge, this narrate is the first published case report of possibly the largest papillary thyroid carcinoma in SO with metastasis to the opposite ovary. Ethics approval and consent to publish were provided (Medical Research Centre review board, IRB, #16024/16, Hamad Medical Corporation, Doha, Qatar).

## Case presentation

A 42-year-old Indonesian female, presented at Hamad General Hospital in Doha, Qatar complaining of an on and off lower abdominal pain mainly in the right iliac fossa. She had a normal delivery 15 years ago, had regular menstrual cycles, and no previous medical illnesses.

### General examination

She was vitally stable, with no significant lymphadenopathy or pedal edema. Abdominal examination revealed midline palpable firm mass with mild tenderness. The mass arose from the pelvis, extending 2 cm below the umbilicus. There was no ascites. Complete blood picture, renal and liver function tests were normal except for hemoglobin of 11.7 g/dl, and CA 125 was elevated (251 KU/L).

### Investigations

**Fig. 1:**
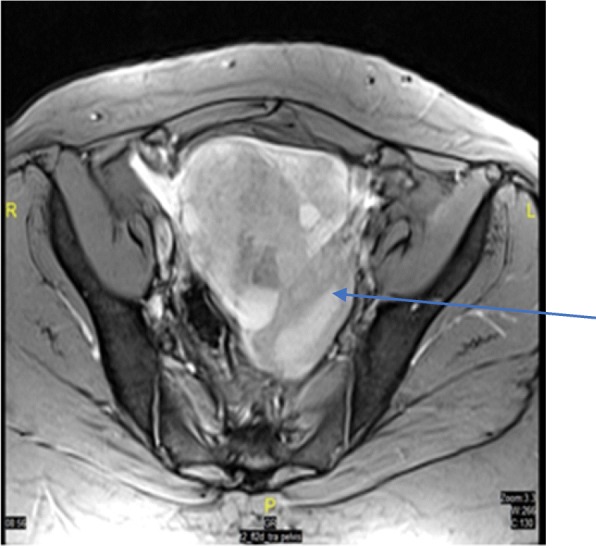
Transverse T2 MRI section. The section shows well-defined complex lesion (arrow) with solid and cystic contents in the pelvis, extending on either side of the midline reaching to both sides of adnexa and measuring 13 × 9.4 × 8.1 cm. Ovaries are not seen separately from the lesion. Uterus shows mild deviation to the left side due to pressure effect from the mass. No obvious lymph nodes or signs of metastasis

Abdominal ultrasound showed a large solid cystic mass in the right adnexa region, reaching the midline (≈6 × 13 cm) with mild vascularity in the solid component. Both ovaries were not separately visualized. There was mild left hydrosalpinx and mild ascites. Transvaginal ultrasound did not show the left ovary, but the right ovary was visualized separately (2.5 × 2.1 cm) and confirmed the presence of complex solid cystic mass in the middle of the pelvis. The mass (13.5 × 9.8 cm) extended to the left adnexa, with cystic area (9.2 × 5.9 cm) and a solid component (9.1 × 7 cm) that had increased vascularity. Further chest/abdomen/pelvis CT and MRI (Fig. 1) confirmed the size and solid/ cystic nature of the mass and showed no metastatic lesions, and also deviation of uterus to the left side.

### Management

**Fig. 2:**
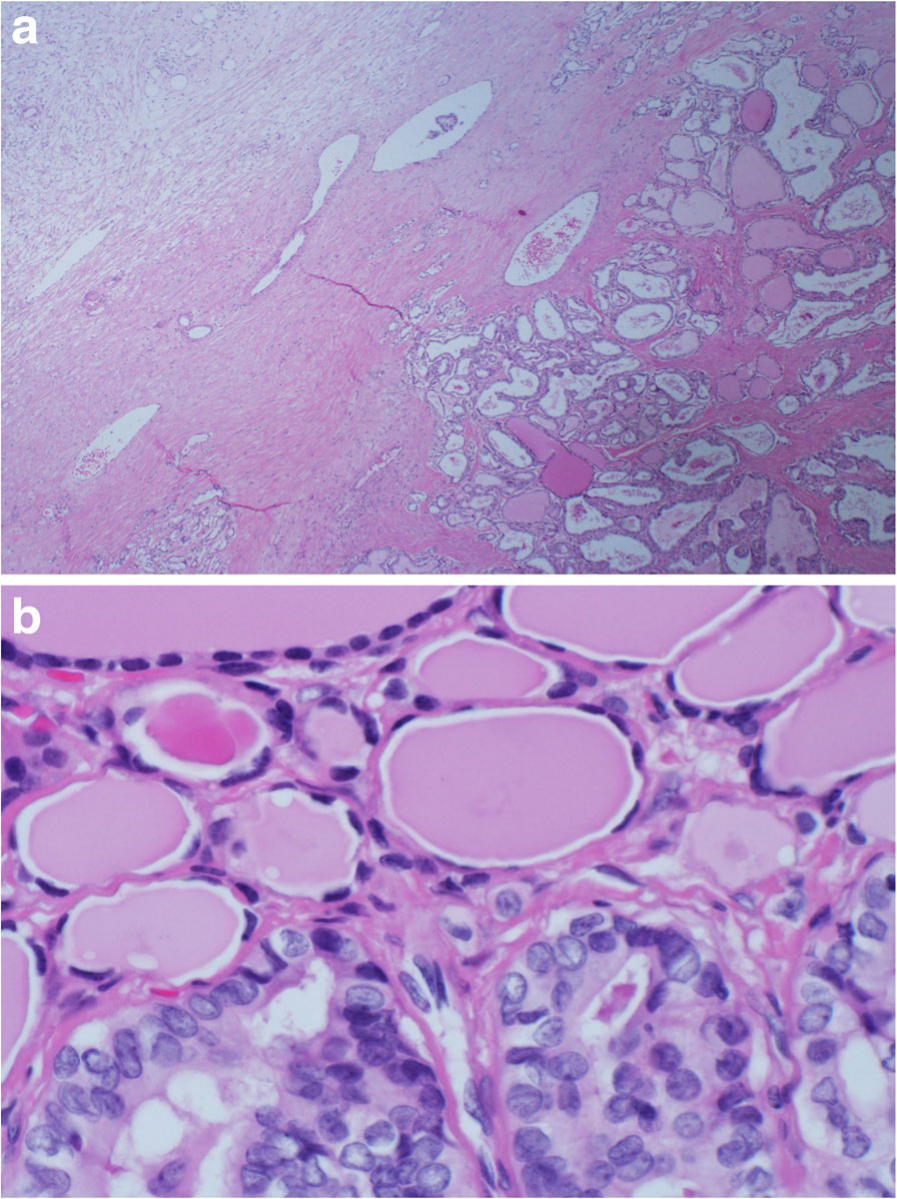
Low and High power hematoxylin and eosin-stained section. **a** Low power hematoxylin and eosin-stained section (4×) demonstrates thyroid follicles of papillary carcinoma arising in benign thyroid follicles of SO. **b** High power hematoxylin and eosin-stained section (60×) demonstrates papillary thyroid carcinoma with follicular pattern. Nuclear features including nuclear groves, clearing, overlapping and enlargement, consistent with papillary thyroid carcinoma arising in a SO

**Fig. 3:**
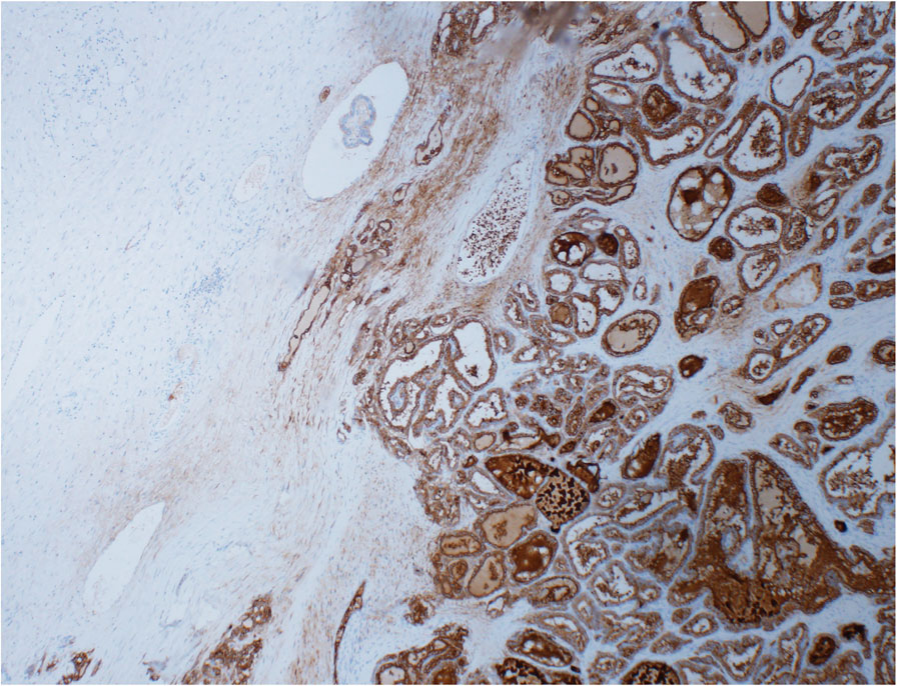
Thyroglobulin immunohistochemical stain. Low power thyroglobulin immunohistochemical stained section (4×) highlights the thyroid tissue in a background of ovarian tissue with SO

The patient’s clinical picture was discussed at our gynecologic multidisciplinary meeting and total abdominal hysterectomy (TAH), bilateral salpingo-oopherectomy (BSO) and lymphadenectomy were decided. Patient underwent TAH + BSO plus infracolic omentectomy. During surgery, a freely mobile left ovarian mass was found with irregular surface and intact capsule. Right adnexa and uterus were normal. Patient had a smooth post-operative recovery and was discharged. Microscopic examination revealed an 11.0 cm left ovarian papillary thyroid carcinoma arising in SO (Figs. 2 and 3), with metastatic papillary thyroid carcinoma to the right ovary. No malignancy was found in right fallopian tube, uterus or cervix and there were negative lymph nodes. Following the histopathology results, patient had thyroid function tests (TSH, free T4, thyroglobulin) that were all normal. Thyroid ultrasound revealed 7 × 11 mm complex nodule, a 6 × 6 mm complex nodule and a 3 × 4 mm cyst in the left thyroid lobe. No lesions were observed in the right thyroid lobe. The patient’s clinical findings were discussed at our thyroid multidisciplinary meeting where total thyroidectomy and radioactive iodine therapy were decided; however the patient refused further surgical management, and was lost to follow up as she left the country.

### Pathologic findings

Upon histopathologic examination, a papillary thyroid carcinoma was identified arising in SO tumor (11.0 cm in greatest dimension) of the left ovary (Figs. 1, 2a and b), and a small metastatic focus measuring 0.1 cm in the right ovary. There was no malignancy in right fallopian tube, uterus or cervix and negative lymph nodes. Thyroglobulin immunohistochemical stained section highlighted the thyroid tissue in a background of ovarian tissue with SO, and confirmed the origin from thyroid tissue (Fig. 3). AJCC Pathologic tumor staging was p T1b and FIGO stage was IB.

## Discussion

SO is an uncommon ovarian tumor with < 5% malignancy, 5–23% metastasis, and 7.5–35% recurrence rates [3, 6, 15, 20]. Our comprehensive literature review of MSO, details a wide range of parameters in relation to MSO that include: size, histopathological categories, type of gynecological surgery, thyroid gland workup and management, and MSO follow up and recurrence (Table 1).

**Table 1:** Case studies of malignant Struma Ovarii

Study*	Country	Tumor		Type of Gynecological Surgery	Thyroid Workup	Thyroid Nodule	Thyroid Management	Follow up	Recurrence
		Type	Size (mm)						
Middelbeek 2017 [29]	USA	PTCF	12	LBSO	^a^	^a^	HT then TT	^a^	^a^
Pineyro 2017 [30]	Uruguay	PTCF	4	Right ovarian cystectomy, left adnexectomy	TFT Normal U/S	4x2x4 mm FNA NC	Conservative	Lost follow up	Lost follow up
Fernández 2016 [35]	Spain	PTC	25	UO	U/S HN	1.5 cm FNA BFN	TT, HP PTC, RAI, LT	6 y	Nil
Wei 2015 [44]	USA	PTCF (8 cases)	1–42	–	–	–	TC	1 m-11 y	–
		PTC (2 cases)	4–30	–	–	–	–	8–15 y	–
		HDFCO	–	–	–	–	–	17 y	Nil
		PTCT and OM (2 cases)	–	–	–	–	–	NC	–
Monti 2015 [45], Goffredo 2015 [8]	Italy USA	PTC 68 (HP NC)	– mean 52.8 (1–200)	UO UO, BO,oophorectomy and omentectomy, debulking surgery	U/S, TFT, TgAb NC	Nil NR	Prophylactic TC, RAI TT	NC 2 m- 34 y (mean 8 y)	– –
Kumar 2014 [27]	India	PTCF	–	UO,TAH, omentectomy, appendectomy	TFT, U/S	Nil	TT, HP lymphocytic thyroiditis	1 y	Nil
Mardi 2013 [46]	India	PTCT	–	Cystectomy	–	–	–	6 m	Nil
Leite 2013 [31]	Portugal	PTC	–	USO	–	–	Complete thyroidectomy, HP PTCF	2 y	Nil
Meringolo 2012 [47]	Italy	PTC	3	Monolateral annessectomy	TFT, TgAb, TPO ab	Yes, FNA benign	LT	–	–
Barrera 2012 [24]	Philippines	PTC	–	TAH BSO	TFT, U/S, HNs	No FNA done	RIA, LT	6 m	Nil
Stanojevic 2012 [32]	Japan	PTCF	10	USO, contralateral cystectomy(HP benign)	TFT, Tg, TgAb U/S	6 × 4 mm	Patient planned for FNA and TT	–	–
O’Neill 2012 [33]	Ireland	PTC	–	USO	NC	–	TT, HP normal, RAI	–	–
Jean 2012 [28]	USA	PTC	25	BSO, peritoneal biopsy, lymph node sampling	TFT, U/S	2.7 cm nodule	TT (HP benign), RAI	2y	Nil
Tanaka 2011 [26]	Japan	PTCF	30	Total hysterectomy + USO	–	–	–	14 m	Nil
Shaco-Levy 2010 [48]	USA	FTC	–	–	–	–	–	–	Yes in 15 patients^b^
		PTC (24 cases, 4 re classified as AC)	All NR except one (2)	–	–	–	RAI	–	
		FA (60)		–	–	–	RAI	–	
Sibio 2010 [25]	Italy	PTC	1	Hysterectomy, UA, peritoneal implants removal, LL	Patient had previous Total Thyroidectomy			7 y	Nil
Coyne 2010 [36]	USA	PTCF	–	Unilateral ovarian cystectomy	TFT, U/S, CT	Patient planned for final pregnancy followed by TT + RAI		–	–
Robboy 2009 [18]	USA	FTC (3 cases)	–	UO /TAH BSO/tumor debulking	–	–	Thyroidectomy/ biopsy in 14 patients	25 y; 10 y survival 89, 84% at 25 y	Yes in 10 patients^c^
		PTC (20 cases)	–	“	“	“	“		
		PTCF (1 case)	–	“	“	“	“		
		PTC + MA (4 cases)	–	“	“	“	“		
		Adenomatous patterns (58)	–	“	“	“	“		
Garg 2009 [22]	USA	PTC (2 cases) PTCF(4 cases) PTCF and PTC Bilateral PTCF Poorly differentiated carcinoma (2 cases)	1.1–80	Cystectomy, USO, TAH BSO, hysterectomy with USO	Radioactive iodine scan, thyroglobulin	–	TT(HP benign) and RAI in two patients,	1 to 14 y	2 cases^d^
Roth 2008 [34]	USA	PTC (3 case)	–	^e^	^e^	^e^	^e^	^e^	^e^
		FTC poorly differentiated (1 case)	–	^e^	^e^	^e^	^e^	^e^	^e^
Salvatori 2008 [37]	Italy	PTCF	–	^f^	^f^	^f^	^f^	^f^	^f^
Yassa 2008 [3]	USA	PTC	9	–	TSH, TG, TG ab, U/S	1 cm FNA benign	Thyroxine therapy	1 y	none

*AC* Anaplastic carcinoma, *BFN* Benign follicular nodule, *CT* Computerized tomography, *FA* Follicular adenoma, *FTC* Follicular thyroid carcinoma, *HDFCO* (Highly differentiated follicular carcinoma of ovarian origin): tumor involved extra ovarian tissues without nuclear features of PTC, *HN* Hypoechoic nodule, *HP* Histopathology, *HT* Hemithyroidectomy, *LBSO* Laparoscopic bilateral salpingo-oophorectomy, *LL* Locoregional lymphadenectomy, *LT* Levothyroxine, m months, *MNS* Microcarcinoma focus size not specific, *MA* Mucinous adenocarcinoma, *PTC + OM* Primary papillary thyroid carcinoma + ovarian metastasis, *PTC* Papillary thyroid cancer, *PTCF* PTC follicular variant, *PTCT* Tall cell variant, *RAI* Radioactive iodine, *SO* struma ovarii, *TAH BSO* Total abdominal hysterectomy and bilateral salpingo-oophrectomy, *TAH* Total abdominal hysterectomy, *TFT* Thyroid function tests, *TgAb* Anti-thyroglobulin antibody, *TPO ab*: thyroperoxidase antibody, *TT* Total thyroidectomy, *U/S* Ultrasound, *UA* Unilateral adnexectomy, *UO* Unilateral oophorectomy, *USO* Unilateral salpingo-oophorectomy, y years

*Due to space considerations, only first author is cited; “: same as above; –: not reported, cannot be inferred

^a^Patient diagnosed initially as thyroid PTCF, had HT followed by TT, thyroid scan and SPECT (right adnexal mass uptake), histopathology: PTCF within SO suggestive of primary disease not metastatic, radio iodine treatment given postoperative, no recurrence features over 5 years

^b^15 patients with recurrences (11 FA, 4 PTC)

^c^10 patients with recurrences, initial gynecological operation for each is not clear

^d^First patient had left ovarian cystectomy, HP later found to be SO + PTCF. On 3 years follow up right ovarian tumor 2.4 cm detected, during surgery cul de sac and omentum implants found, HP was PTC. Patient then had RAI scan (diffuse uptake in abdomen), TT done, then RAI therapy given. Second patient had left ovarian cyst, ovarian cystectomy done. Caesarian section four years later (uterus, pelvis, cul-de-sac lesions found, TAH BSO done, PTCF lesions), RAI scan done (diffuse uptake in chest/ abdomen), patient had TT + RAI. Also had metastatic liver mass 8 cm (PTCF) that was resected. It is noted that recurrences in both patients occurred with well-differentiated and small foci of their primary tumors

^e^One PTC case had unilateral adnexal excision, paraortic LNs dissection + radiation therapy postoperative. Thyroid workup/ management NC. Follow up/ recurrence NC. One PTC case had right oophorectomy, left ovarian cystectomy and uterine curettage. Thyroid workup/ management NC. Follow up 25 years and patient is well. One PTC case had TAH BSO and pelvic node dissection, died soon after surgery. One poorly differentiated FTC had TAH BSO and peritoneal biopsies, then total TT, RAI and chemotherapy. Died 3years after primary operation

^f^Initial operation was right salpingo-oopherectomy for right ovarian cyst, HP was SO with mature cystic teratoma, patient then had enucleation of left ovarian cyst (HP: PTCF) and multiple biopsies from pink nodules in abdomen and pelvis (HP: endometriosis). Then patient had TT and RAI scan (multiple liver, abdominal, pelvic uptakes), CT and MRI (multiple abdominal/ pelvic nodules). Patient underwent debulking of nodular mass, partial omentectomy and partial excision of ovarian cortex (due to patient’s wish), followed by RAI therapy

Regarding the tumor size of MSO, a range of dimensions (0.1–4.2 cm) has been reported (Table 1), and an analysis of large series of 68 MSO patients observed a mean tumor size of 5.28 cm [8]. To the best of our knowledge, our MSO is the possibly the largest (11 cm) reported MSO with PTC tumour confirmed by histopathology to date. Others found a MSO measuring 20 cm, but did not report the tumor histopathology; hence we are unable to judge their tumor subtype [8]. Our MSO is also first to be reported from the Middle East and North Africa region. Such a large sized tumor is likely to cause pressure effects (as observed in our patient who had deviation of uterus to the left side) (Fig. 1).

As for the relationship between tumour size and metastasis, research [21] reported that a larger sized tumor was associated with higher probability of metastasis. We are in agreement, as our tumour (11 cm) showed PTC metastasis to the contralateral ovary. Nonetheless, it remains to be established whether the relationship between primary tumour size and metastasis is consistent for all MSO. For instance, others [22] reported two patients with metastasis despite their small primary tumors (first was 8 mm MSO tumour with contralateral ovarian metastasis; second comprised multiple small tumour foci in left ovary with metastasis to the liver).

In terms of management of the primary (ovarian) tumour, no standard guidelines exist for treatment of papillary thyroid carcinoma arising in MSO due to its scarcity. TAH + BSO and omentectomy are considered optimal, however due to the permanent infertility associated with this procedure, unilateral salpingo-oopherectomy/unilateral oophorectomy in order to preserve the patients’ fertility is suggested, as more aggressive approaches did not decrease the tumour’s recurrence rate [8, 20, 23]. Our patient received TAH + BSO, in agreement with the published literature [18, 24].

As for recurrence, our primary tumour was in the left ovary with PTC metastasis to the right ovary, but we are unable to report on recurrence as our patient left Qatar (lost to follow up). Treatments for the primary ovarian tumor include: TAH + BSO (considered ideal), with no recurrence over 6 months - 4 years follow up [22, 24]; hysterectomy and unilateral oophorectomy/ unilateral salpingo-oopherectomy with no recurrence over 1–7 years [22, 25–27]; bilateral salpingo-oophrerectomy with no recurrence over 2–5 years [28, 29]; and unilateral oophorectomy/ unilateral salpingo-oophorectomy with no recurrence over 1–25 years [22, 30–35]. Our patient received TAH + BSO that has good reported outcomes [22, 24, 29].

Whilst the role of ovarian cystectomy alone in managing MSO is unclear due to lack of data, ovarian cystectomy alone may be suboptimal as the patient may subsequently present with recurrences/ metastasis. For instance, a patient received ovarian cystectomy for SO with PTCF, but follow up and recurrence were not known [36]; another patient had right salpigo-oopherectomy for SO with mature cystic teratoma and enucleation of left ovarian cyst for PTCF, where multiple metastasis were subsequently found [37]; and two patients who had ovarian cystectomy as initial operations, both subsequently presented with metastasis [22]. Nevertheless, for patients with unilateral adnexal mass (unilateral MSO), both unilateral salpingo-oophrecetomy /unilateral oophorectomy and TAH seem effective. However, careful pre/operative assessment of the contralateral side and consistent post-operative follow up are recommended, as it may also harbor (benign or malignant) SO. Others found contralateral benign SO associated with unilateral SO tumor (not described whether malignant or benign) (4 cases) [18]; contralateral MSO PTCF associated with unilateral benign SO [37]; while both the current study and other reports [22] observed contralateral malignant metastatic deposits from the unilateral primary MSO tumour.

As for management of the thyroid gland itself, debate remains about the role of thyroidectomy and radioactive iodine ablation (T + RIA) for MSO. Most authors support aggressive treatment by surgical removal of the tumour followed by radiotherapy, chemotherapy and radioactive iodine therapy regardless of metastasis at time of diagnosis [4, 6, 38–41] (see also Table 1), and our MDT decision in managing our patient was in agreement with such an approach. Conversely, others hold that surgical removal of the ovarian tumour only is sufficient, and thyroidectomy and radioactive iodine to be undertaken in case of metastases or recurrent disease [42]. Certain SO characteristics may necessitate thyroidectomy and radioactive iodine therapy (e.g. tumor size ≥1 cm, disease outside ovary, or histopathological features of aggressive tumor) [3, 43]. Moreover, MSO may actually increase the risk of additional thyroid cancer [23], where, among 68 MSO, 9% had primary thyroid cancer in the neck, and 67% had invasive thyroid cancer disease [8]. Early genomic instability and gene mutations may provide a common pathogenesis for all papillary thyroid cancers irrespective of their body locations [21, 40].

## Conclusion

This report is a comprehensive literature review of MSO, detailing the sizes and histopathological categories, types of gynecological surgery, thyroid gland workup and management, and follow up and recurrence. Our case report is possibly the largest MSO PTC in the literature. TAH + BSO seems to be best in terms of curative outcome, however, hysterectomy with unilateral salpinogo-oophrectomy/unilateral oophorectomy, bilateral salpingo-oopherectomy and unilateral salpingo-oopherectomy/unilateral oophorectomy seem also effective treatment options. Fertility may be preserved with unilateral salpingo-oopherectomy/ unilateral oophorectomy, as this has great impact on the patient’s psychology and social life. When unilateral removal of adnexal mass in undertaken, the contralateral side should be carefully assessed with surveillance for metastatic MSO. Ovarian cystectomy alone is associated with recurrences/ metastasis. Debate remains as to the association between MSO tumor size and potential extent of metastasis, and about the management of thyroid gland, however, surveillance and thyroid gland work up to detect concurrent thyroid cancer are recommended.

## Abbreviations

AJCC: American Joint Committee on Cancer
CT: Computerized tomography
MDT: Multi disciplinary team
MRI: Magnetic resonance imaging
MSO: Malignant struma ovarii
PTC: papillary thyroid carcinoma
PTCF: papillary thyroid carcinoma follicular variant
SO: Struma ovarii
T + RIA: thyroidectomy and radioactive iodine ablation
T4: Thyroxine
TAH BSO: Bilateral salpingo-oopherectomy
TAH + BSO: Total abdominal hysterectomy and bilateral salpingo-oopherectomy
TAH: Total abdominal hysterectomy
TSH: Thyroid-stimulating hormone
